# Video vs direct laryngoscopy in the ICU: are we asking the right question?

**DOI:** 10.1186/s13054-017-1701-6

**Published:** 2017-05-16

**Authors:** Michael Chaim Sklar, Stephen Lapinsky

**Affiliations:** 1 Department of Anesthesiology, University of Toronto, Toronto, ON Canada; 2 Mount Sinai Hospital and Interdepartmental Division of Critical Care, University of Toronto, Toronto, ON Canada

We read with interest the recent publication in *JAMA* by Lascarrou et al. [1] of video vs direct laryngoscopy for tracheal intubation in intensive care unit (ICU) patients. We are eager to raise an important issue absent from the discussion and its accompanying editorial [2]: the danger of administering drugs to facilitate intubation in hypoxemic and hypotensive patients which may induce apnea and exacerbate hypotension. The mode of laryngoscopy may not matter but the conditions used to facilitate it most certainly do. We advocate for an awake intubation attempt in the critically ill.

Endotracheal intubation in the operating room (OR) and the ICU are different procedures, but this is not always recognized. The ICU patient should be evaluated as a physiologically difficult airway [3], in contrast to the traditional difficult airway evaluated in the OR. ICU intubation usually occurs in an unstable patient often with a short period of time to allow for evaluation and planning, and in an environment not always ideally suited to airway management.

Inducing apnea for ICU intubations is of major concern for several reasons. Unlike the elective patient who can withstand 6–8 minutes of apnea if preoxygenated, the arterial saturation rarely rises with preoxygenation and apnea induces rapid desaturation [4]. Compensatory hyperventilation is lost and acidosis worsens during apnea.

Lascarrou et al.’s [1] patients were severely hypoxemic (median P_a_O_2_/FiO_2_ < 100 mmHg) and in shock (mean serum lactate >3 mmol/L), yet patients received “general anesthesia” with hypnotic agents and neuromuscular blockers. So-called “hemodynamically stable” agents such as ketamine and etomidate still induce hypotension with very deep sedation, and the administration of rapidly acting paralytics may still be fraught with danger, especially when used by nonexperienced intubators. Indeed 84% of intubations in this study were performed by nonairway experts, with <10% performed by anesthesiologists. The rate of severe life-threatening complications was approximately 12%.

Intubating the ICU patient while maintaining spontaneous respiration reduces the risk of worsening hypoxemia and hypotension, and allows time for expert personnel to assist if initial attempts fail. In healthy patients, direct laryngoscopy is usually possible with minimal sedation [5]. The ICU patient, due to sepsis, hypoxemia, or hypercapnia, will have a reduced level of awareness and can usually be intubated with minimal sedation and topicalization.

The accompanying editorial [2] concludes that optimal care should recognize blind spots when caring for the critically ill; perhaps the blind spot is not the laryngoscope, but assuming that ICU intubations require general anesthesia.

## Abbreviations

ICU: intensive care unit
*JAMA*: *Journal of the American Medical Association*
OR: operating room
